# Age-related slowing of sensorimotor reaction time in masters track and field athletes

**DOI:** 10.1371/journal.pone.0360641

**Published:** 2026-11-11

**Authors:** Yanbing Zhou, Natalie N. McLaurin, Jonas Böcker, Philip D. Chilibeck, Tanja Taivassalo, Sean C. Forbes, Dominik Pesta, Jörn Rittweger, Hirofumi Tanaka

**Affiliations:** 1 Department of Kinesiology and Health Education, The University of Texas at Austin, Austin, Texas, United States of America; 2 Institute of Aerospace Medicine, German Aerospace Center (DLR), Cologne, Germany; 3 College of Kinesiology, University of Saskatchewan, Saskatoon, Canada; 4 Department of Physiology and Aging, University of Florida, Gainesville, Florida, United States of America; 5 Department of Physical Therapy, University of Florida, Gainesville, Florida, United States of America; 6 Medical Faculty, Cologne Excellence Cluster on Cellular Stress Responses in Aging-Associated Diseases (CECAD), University of Cologne, Cologne, Germany; 7 Cologne Excellence Cluster on Cellular Stress Responses in Aging-Associated Diseases (CECAD), University of Cologne, Cologne, Germany; 8 Faculty of Sport Science, Chulalongkorn University, Bangkok, Thailand; Universidade Federal do Rio Grande do Sul, BRAZIL

## Abstract

**Introduction:**

Masters athletes offer a powerful model for understanding how lifelong training shapes age‑related changes in reaction time (RT), a key indicator of sensorimotor performance.

**Purpose:**

This cross-sectional study examined RT in masters track and field athletes across age, sex, RT tasks of varying complexity, and event disciplines (endurance, power, sprint)

**Methods:**

A total of 182 competitors (82 females) from the 2025 World Masters Athletics Championships completed four RT tasks using a wireless light-based testing system.

**Results:**

RT increased progressively with age across all tasks, with the slowest responses consistently observed in athletes ≥80 years old. Female athletes demonstrated slower RT than males, and RT increased systematically with task complexity. Sprint athletes showed faster RT than endurance athletes overall, although event‑specific differences did not persist within age groups. Age was positively associated with RT in all tasks (r = 0.41–0.53), with similar correlation strengths between sexes. Quadratic age effects emerged selectively in male athletes for two-hand simple and step-choice tasks, suggesting accelerated slowing at older ages in tasks requiring greater motor coordination. Linear regression models indicated that RT increased by approximately 3.3–3.6 ms per year of age after adjusting for sex, task, and event discipline, while body composition variables were not significant predictors.

**Conclusions:**

Although lifelong training is associated with better sensorimotor performance, slower RT remains evident in masters athletes, with consistent sex differences and minimal influence of event discipline.

## Introduction

Reaction time (RT) is a fundamental component of sensorimotor performance, reflecting the speed with which an individual detects a stimulus, processes information, and initiates a motor response [1]. Because RT integrates sensory input, central neural processing, decision-making, and motor execution, tasks of varying complexity are widely used to assess neuromuscular integrity and cognitive-motor function [2,3]. Age-related declines in central processing speed and synaptic efficiency, along with alterations in motor unit recruitment [4,5], collectively contribute to slower RT in older adults [6] even in the absence of age-related diseases [6]. The importance of RT extends beyond sport-specific performance as it is closely linked to functional independence in older adults, such as reduced fall risk [7].

Masters athletes provide a unique and informative model for examining how lifelong physical training interacts with the aging process [8]. Unlike the general population, these athletes maintain structured, high-volume exercise across decades [9], lifestyle behaviors associated with preserved brain structure and function, enhanced neuroplasticity, and improved neuromuscular and motor performance [10–12]. Although habitual training may attenuate age-related physiological deterioration [13], it does not fully prevent reductions in brain volume, corticospinal excitability, and motor unit remodeling, as these changes remain evident even in highly trained older adults [14–16].

Sex differences in sensorimotor RT are well-documented in the general population, with males typically showing faster RT than females [17–19] due to a combination of physiological and sociocultural factors [17,18]. Whether these differences persist, diminish, or shift in highly trained older adults remains unclear. Additionally, RT may vary across athletic disciplines that impose distinct neuromuscular and cognitive demands. Sprint and power events rely heavily on rapid neural activation and explosive motor output, whereas endurance events emphasize sustained neuromuscular efficiency. Although preliminary studies suggest better RT performance in masters athletes than in age-matched sedentary peers [20,21], the existing studies in masters athletes are limited by relatively small sample sizes, narrow age ranges, and insufficient representation across event types.

A clear understanding of how age, sex, task complexity, and event disciplines shape RT in masters athletes would provide valuable insight into the mechanisms of preserved sensorimotor performance in aging. Therefore, the primary aim of this study was to examine the associations of age, sex, task complexity, and event disciplines with RT in a large cohort of masters track and field athletes. We hypothesize that RT would slow with advancing age despite lifelong exercise training, that males would exhibit faster RT than females, and that sprint and power athletes would outperform endurance athletes.

## Materials and methods

### Study design and participants

All masters athletes recruited in this cross-sectional study were from the 2025 World Masters Athletics Indoor Championships in Gainesville, Florida, from 23/03/2025–30/03/2025. Written informed consent was obtained from all participants before participation. The study protocol was approved by the Institutional Review Board at the University of Florida (IRB 202401807). All procedures were conducted in accordance with the Declaration of Helsinki. Eligibility criteria included registered participation in the 2025 World Masters Athletics Indoor Championships, age ≥ 35 years in accordance with World Masters Athletics regulations, and not currently pregnant or lactating. Athletes were excluded if they reported acute illness or injury that prevented participation in the study procedures or if informed consent was not provided.

Athletes were classified into five age groups defined a priori based on established master athletics age classifications: 35–49, 50–59, 60–69, 70–79, and ≥80 years old. Based on their self-rated best athletic competition events, athletes were arbitrarily grouped into three sub-disciplines: sprint (60 m, 200 m, 400 m running, and 60 m hurdles), endurance (800 m, 1500 m, 3000 m running, 6 km cross-country (>70 years of age), 8 km cross-country (<70 years of age), and 10 km road race), and power (hammer throw, weight throw, shot put, discus throw, javelin throw, high jump, long jump, and triple jump) as previously described [22,23]. Some athletic events could arguably fall into different sub-disciplines (e.g., long jump into Sprint, 400m run to Endurance). However, moving these events to different sub-disciplines did not influence our overall results.

### Participant characteristics

Participants reported demographic information, including age, sex, highest education level, and the event discipline in which they achieved their best performance via survey. Height was measured using a stadiometer (Seca, Vogul & Halke, Hamburg, Germany). Body weight and body composition, including skeletal muscle mass, fat mass, and body fat percentage, were assessed using a bioelectrical impedance analysis device (InBody 770, InBody Co., Seoul, South Korea).

### Reaction time assessments

Participants were instructed to complete four RT tasks, including three hand tests (one-hand simple, two-hand simple, and one-hand choice) and one foot test (step choice RT), using the FitLight Trainer™ system (FitLight Sports Corp., Canada). Fig 1 provides a schematic representation of the experimental setup. All tests were conducted with participants standing upright and facing the FitLight sensors. Sensors were configured to detect responses using both impact and proximity detection with low sensitivity to minimize inadvertent activations. As measured by the FitLight system, RT reflects the total elapsed time between stimulus (light) onset and physical deactivation of the target, thereby encompassing both the premotor/central processing component and the motor execution component of the response. Testing was conducted in a dedicated, reserved room adjacent to the athlete registration area throughout the day. Fatigue was not formally assessed, but most athletes came in on their off-competition day.

**Fig 1:**
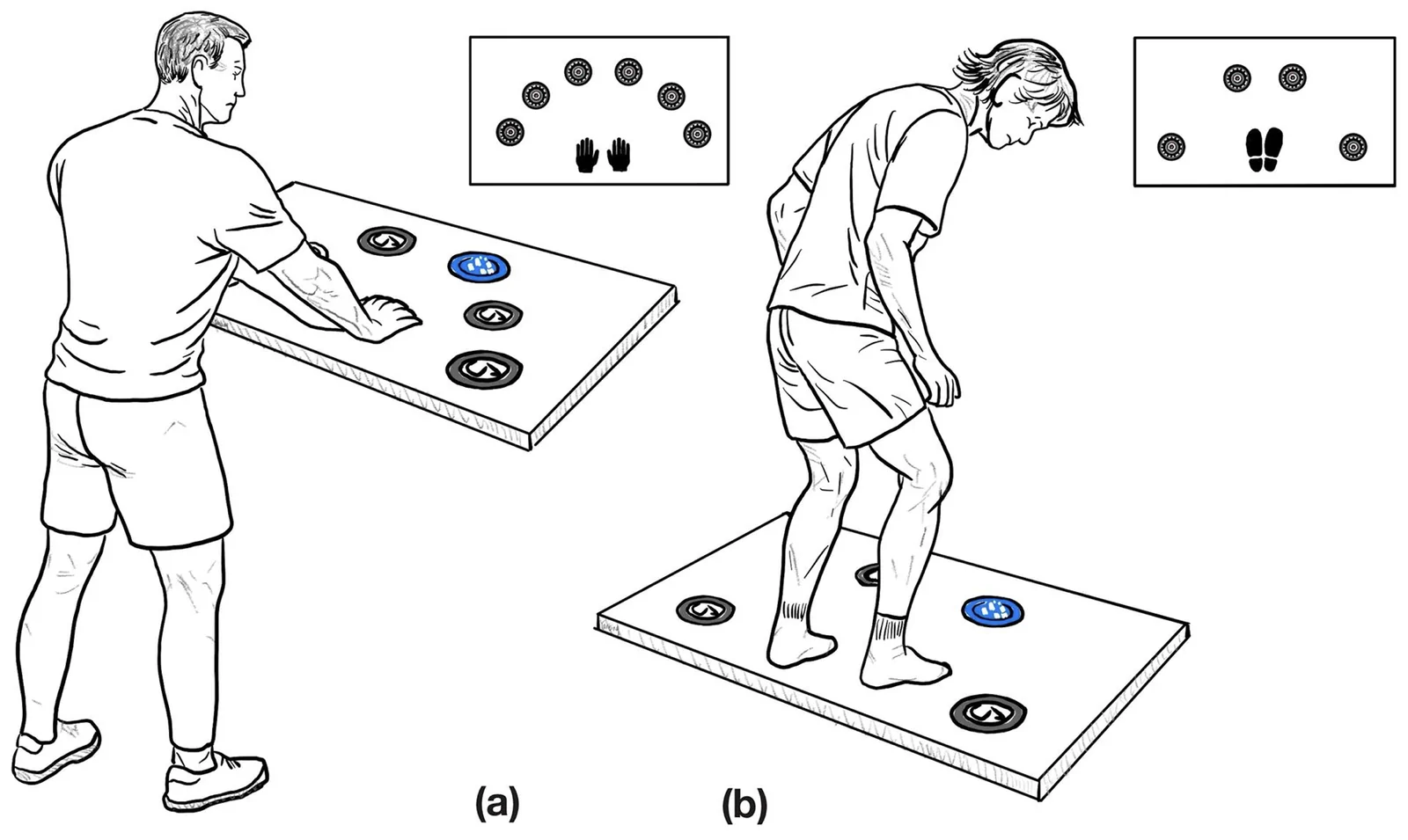
Reaction time tests, including hand (a) and step (b) layout.

Instructions were provided prior to each task. Participants completed a familiarization trial prior to testing to ensure comprehension of task instructions. Each hand test was conducted on a table using six light sensors arranged in a semicircle, spaced 20 cm apart and 40 cm from the center of the semicircle. For the one-hand and two-hand simple tasks, participants deactivated the illuminated target light as quickly as possible for a total duration of 60 s. Only one light was activated/illuminated randomly at any given time. A fixed delay of 1 s preceded each stimulus, and a timeout of 2 s was applied. In the one-hand simple task, participants were instructed to respond consistently with their dominant hand when deactivating the target light. In the two-hand simple task, participants responded by either hand to deactivate the target light. The one-hand choice task was performed using an advanced sequence configuration with a total duration of 60 s. Two lights of different colors (blue and green) were presented randomly at any testing time, and participants responded by using their dominant hand to deactivate the illuminated blue light as quickly as possible.

The step choice RT task was assessed with four lights placed securely on a firm surface on the ground and positioned within a rectangular stepping area to ensure consistent stepping height across targets, adapted from a previously validated protocol [24]. Participants responded by stepping toward the illuminated light and deactivating it with their left foot when one of the two lights on the left illuminated, or with their right foot when one of the two lights on the right illuminated. One light was randomly activated at a time for a total of 20 light activations. No timeout was applied for the step choice task, and an inter-stimulus delay of 2 s was used.

### Statistical analyses

A priori sample size estimation was conducted using G*Power 3.1 (Heinrich-Heine-Universität Düsseldorf, Düsseldorf, Germany). For the main effect of age group on RT (F tests, one-way Analysis of Variance, ANOVA), assuming a large effect size (f = 0.40), α = 0.05, power = 0.95, and five age groups, the required total sample size was 125. When age was treated continuously, a multiple linear regression model was used assuming a medium effect size (f² = 0.15), α = 0.05, power = 0.95, and seven predictors corresponding to age, sex, event discipline, and tasks, yielding a total sample size of 153.

Outliers (defined values beyond ±3 SD of the task-specific mean) were removed for each reaction time task. This resulted in the exclusion of five task-specific data points from three participants: step-choice task data from one participant; two-hand simple task data from another participant; one-hand simple, two-hand simple, and hand-choice task data from a third participant. The removal of these outliers did not influence the overall study results. Levene’s test was conducted to test the assumption of homogeneity of variance. The Shapiro-Wilk test was conducted to assess the normality of all variables. Skewness was tested for the two-hand simple (0.54), one-hand simple (0.50), hand-choice (0.46), and step-choice (0.44) tasks, which were within ±1, indicating approximate normality and feasibility for parametric tests. The Independent samples t-tests were used to compare differences by sex. The Levene’s test was conducted to assess the assumption of homogeneity of variances. One-way ANOVA was performed to evaluate the overall differences by age groups. Two-way ANOVA (age–sex, age–event discipline) was conducted to test the interactions of age group and event discipline or sex. Following a significant F-statistic, the Tukey–Kramer post-hoc analysis was conducted to evaluate specific group differences while adjusting for unequal group sizes. A sensitivity analysis was performed by re-running the primary age-group ANOVAs with outliers retained to assess whether their exclusion influenced the results. Chi-square test with standardized residuals was performed to assess differences in categorical variables. Pearson correlation analyses were conducted to evaluate linear associations between age and RT across the four RT tasks and sexes, with r as the correlation coefficient (small effect: r = 0.10; medium effect: r = 0.30; large: r = 0.50) [25]. Benjamini-Hochberg correction was used to adjust for multiple comparisons and to control the False Discovery Rate. Differences in correlation strength between sexes were assessed using Fisher’s r-to-z transformation. To explore potential nonlinear age effects, sex-stratified linear and quadratic regression models were used to examine the relationship between age and RT. Multiple linear regression models were constructed to evaluate independent predictors of RT. All statistical analyses were conducted in R 2025.05.1 + 513 (R Foundation for Statistical Computing, Vienna, Austria). The statistical significance was set at p < 0.05 for all comparisons.

## Results

Participant characteristics are shown in Table 1. A total of 182 participants comprising 82 females and 100 males were included in the analysis. Males were taller, heavier, and had higher BMI and body fat percentage than females (all p < 0.001). Age and event-discipline distributions were similar across sexes (all p > 0.05), except that more males were postgraduate than females (p = 0.011). RT differed by task complexity. One-hand simple (0.76 ± 0.08 [0.75, 0.77] s) and two-hand simple (0.75 ± 0.08 [0.74, 0.76] s) were comparable whereas one-hand choice (0.85 ± 0.09 [0.84, 0.86] s) and step choice (0.95 ± 0.11 [0.93, 0.97] s) were significantly slower (all p < 0.01). Female athletes demonstrated slower (longer) RT than male athletes across all tasks (all p < 0.05). RT differed significantly by event discipline across all four tasks (all p < 0.05), with sprint athletes demonstrating the fastest RT and endurance athletes the slowest, one-hand simple (Endurance: 0.78 ± 0.07 [0.76, 0.80] s; Power: 0.76 ± 0.08 [0.73, 0.79] s; Sprint: 0.74 ± 0.07 [0.72, 0.76] s), two-hand simple (Endurance: 0.77 ± 0.08 [0.75, 0.79] s; Power: 0.76 ± 0.08 [0.73, 0.78] s; Sprint: 0.73 ± 0.07 [0.71, 0.74] s), one-hand choice (Endurance: 0.87 ± 0.10 [0.85, 0.90] s; Power: 0.85 ± 0.10 [0.82, 0.88] s; Sprint: 0.82 ± 0.08 [0.80, 0.84] s), and step-choice (Endurance: 0.98 ± 0.11 [0.95, 1.00] s; Power: 0.94 ± 0.09 [0.91, 0.97] s; Sprint: 0.92 ± 0.12 [0.89, 0.95] s). The distribution of participants stratified by age and event discipline is presented in S1 Fig and S1 Table.

**Table 1:** Selected participant characteristics.

Variable	All	Females	Males	P value
N (%)	182	82 (45)	100 (55)	
Age (year)	62 ± 13	61 ± 13	63 ± 14	0.311
Height (cm)	168 ± 9	162 ± 6	172 ± 8	<0.001
Body weight (kg)	67.7 ± 14.4	60.4 ± 10.5	73.8 ± 14.4	<0.001
BMI (kg/m^2^)	24.0 ± 4.1	23.0 ± 3.9	24.7 ± 4.2	0.004
Body fat (%)	21.0 ± 8.7	19.3 ± 8.3	22.4 ± 8.9	0.022
Sporting event discipline				
Endurance	67	32 (48)	35 (52)	0.714
Power	41	21 (51)	20 (49)	0.876
Sprint	70	27 (39)	43 (61)	0.056
Education				
Secondary or below	22	11 (50)	11 (50)	1.000
Undergraduate	68	35 (51)	33 (49)	0.808
Postgraduate	74	26 (35)	48 (65)	0.011
Reaction time (s)				
One-hand simple	0.76 ± 0.08	0.78 ± 0.08	0.74 ± 0.07	0.002
Two-hand simple	0.75 ± 0.08	0.77 ± 0.08	0.74 ± 0.08	0.020
One-hand choice	0.85 ± 0.09	0.87 ± 0.09	0.84 ± 0.09	0.019
Step choice	0.95 ± 0.11	0.98 ± 0.11	0.92 ± 0.11	<0.001

Data are means ± SD or n (%).

RT progressively slowed with advancing age in all four RT tasks, with the largest slowing consistently observed in athletes aged ≥80 years (Fig 2). In all four RT tasks, the ≥ 80 year group had slower RT than the 35−49, 50−59, and 60−69 year groups (all p < 0.01), and slower than the 70−79 year group in three of the four tasks (simple one-hand, simple two-hand, step-choice; all p < 0.01). The 35−49 year group had the fastest RT overall. The age group effect was large across all tasks (partial η² = 0.22–0.24). Sex differences were most consistent in the 60−69 year group, where females showed slower RT than males across all four tasks (all p < 0.05; d = 0.60–0.64 [0.05, 1.19]). Significant sex differences were also present in the 50−59 year group for the simple one-hand (d = 0.83 [0.22, 1.43]) and step-choice (d = 0.64 [0.04, 1.25]) tasks, and in the 70−79 year group for the simple two-hand (d = 0.79 [0.09, 1.49]) and step-choice (d = 0.73 [0.03, 1.43]) tasks. The overall sex effect was small-to-medium (partial η² = 0.06–0.10). Sprint athletes demonstrated faster RT than endurance athletes overall in the hand-choice (d = 0.66 [0.29, 1.03]) and step-choice (d = 0.56 [0.19, 0.93]) tasks (partial η² = 0.05–0.07). Power athletes generally fell between sprint and endurance athletes, not differing significantly from either overall (all p > 0.05). No age-discipline interaction was observed in either task. A sensitivity analysis was performed to assess whether their exclusion influenced the results. With outliers retained, the age-group effect remained highly significant in all four tasks (all p < 0.01), confirming our findings are robust to their inclusion.

**Fig 2:**
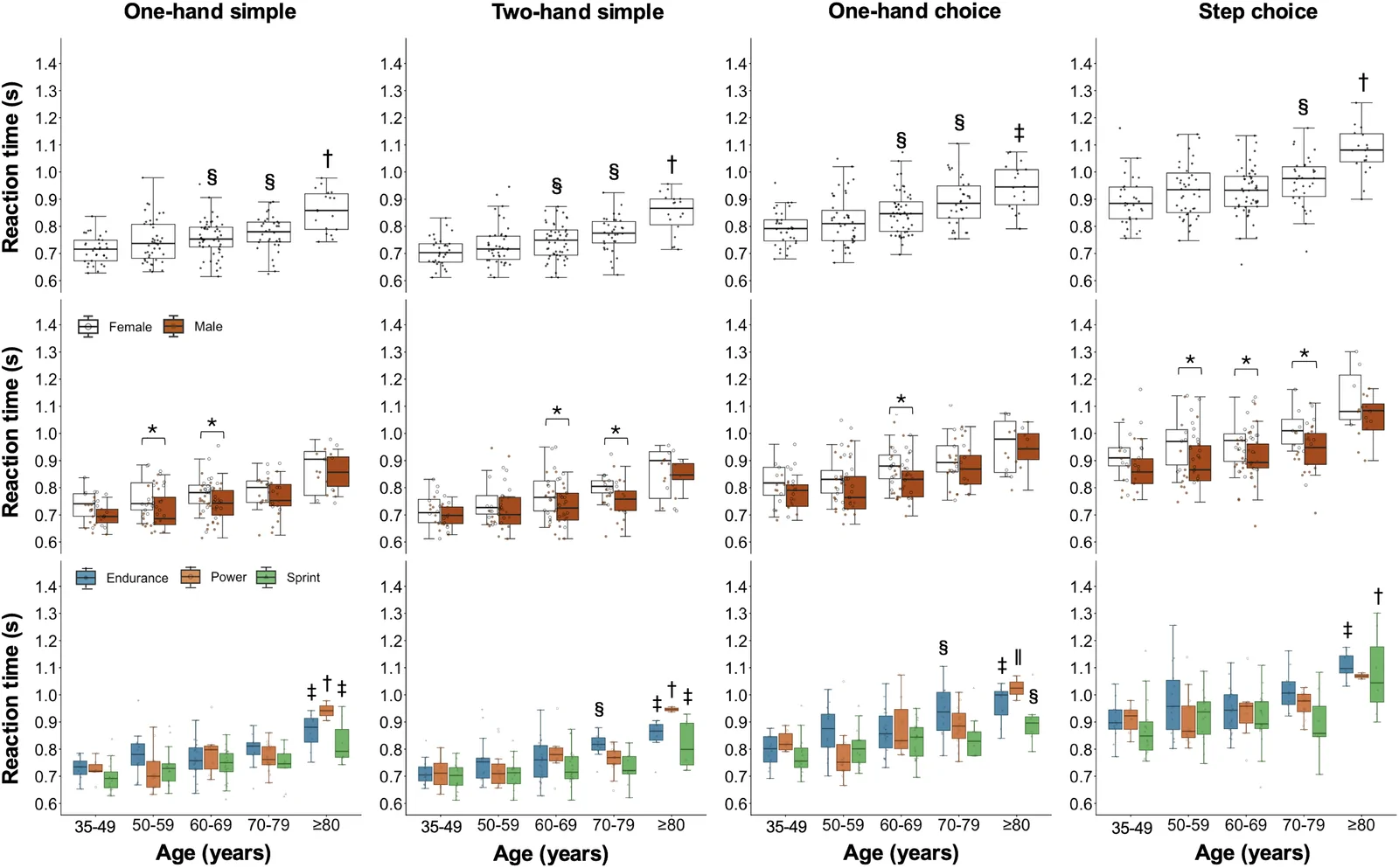
Reaction time stratified by age, sex, and sporting discipline in masters athletes. * p < 0.05 between sexes within the same age group. **†** p < 0.05 between age group ≥80 and all other age groups (35–49, 50–59, 60-69, 70-79). **‡** p < 0.05 vs. age groups of 35–49, 50-59, and 60-69. **‖** p < 0.05 vs. age groups of 35–49 and 50-59. **§** p < 0.05 vs. age group of 35–49.

Pearson correlations between age and RT are shown in Fig 3. Age was positively associated with the one-hand simple (r = 0.49, p < 0.001), two-hand simple (r = 0.50, p < 0.001), choice hand task (r = 0.53, p < 0.001), and step choice tasks (r = 0.41, p < 0.001), with slightly stronger age-related associations observed in the hand-based tasks compared with the step choice task. When stratified by sex, the age–RT associations were consistently positive and statistically significant in both sexes (all p < 0.05). However, the correlations were slightly stronger in males (r = 0.54–0.60) than in females (r = 0.45–0.51) for the hand-based tasks. Fisher’s r-to-z tests showed no significant sex differences in correlation strength (all p > 0.05). There were significant quadratic age effects in the two-hand simple (p = 0.023) and step choice task (p = 0.039) solely in male athletes, but not in female athletes (Fig 3), suggesting that male RT slowing may accelerate at older ages in tasks involving greater motor coordination or whole-body responses, whereas the largely linear patterns observed in the other tasks indicate a relatively steady age-related decline in RT across the adult lives. Discipline-stratified analysis is shown in S2 Fig. To account for multiple comparisons, female and male quadratic effects within each reaction time task were corrected, after which the male-specific curvature effect remained significant only for the two-hand simple reaction time task (adjusted p = 0.046), besides the one-hand simple (adjusted p = 0.195), one-hand choice (adjusted p = 0.770), and step choice (adjusted p = 0.079). Female athletes showed predominantly linear age‑RT relations. Within disciplines, quadratic effects appeared only in sprint athletes (male two‑hand simple: p = 0.030; female step‑choice: p = 0.030), whereas endurance and power athletes showed linear patterns across all tasks.

**Fig 3:**
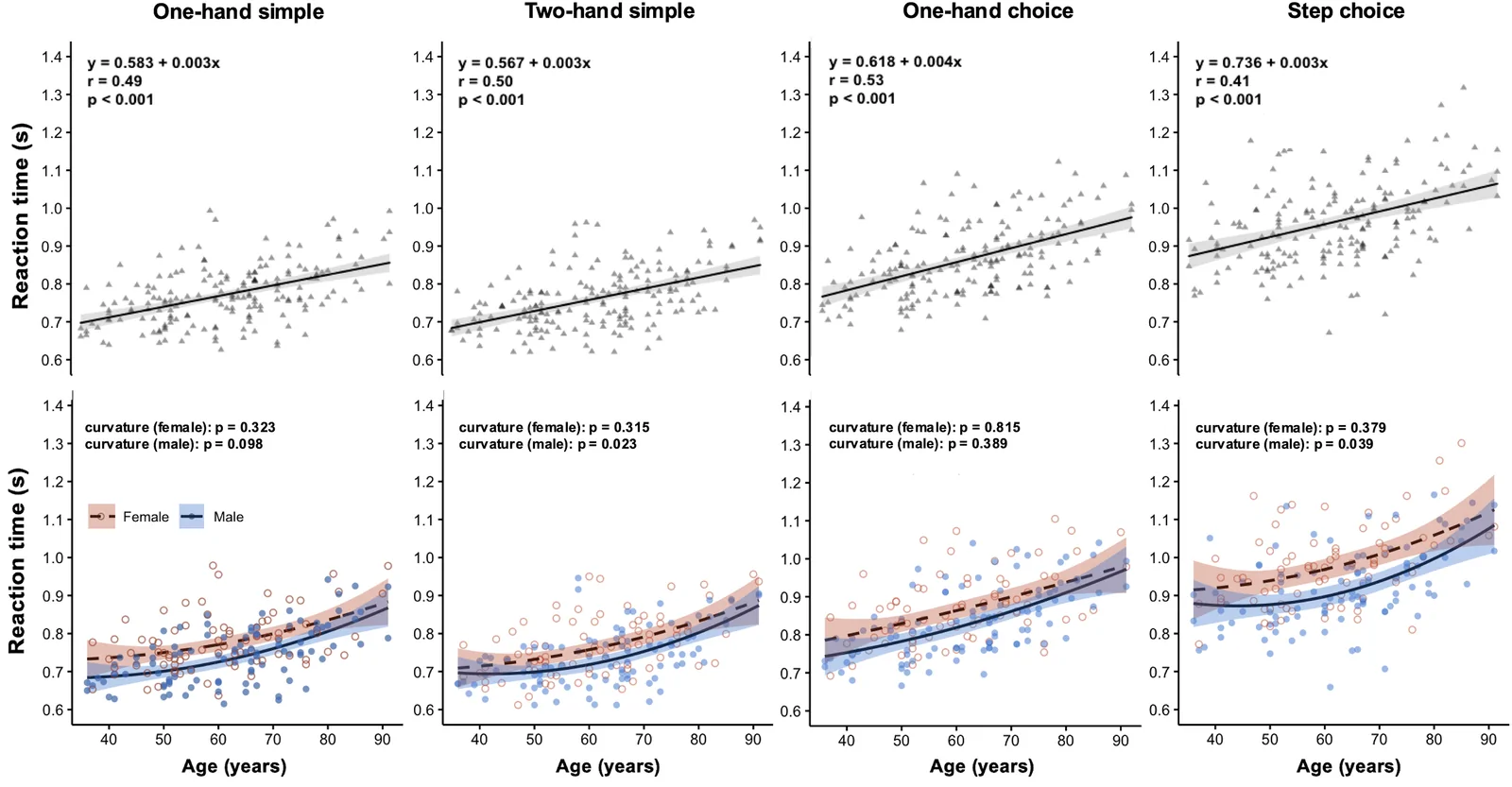
Linear and quadratic correlations between reaction time and age in masters athletes.

A linear age–RT regression model (R² = 0.602, p < 0.001) stratified by RT tasks (Fig 4) indicated that age was significantly associated with RT, indicating that for every 1-year increase in age, RT increases by 3.3 ms per year of age, after accounting for task complexity and sex (β = 0.0033, p < 0.001). Task showed a significant linear trend (β = 0.11, p = 0.011), suggesting RT increased progressively with task complexity. Male athletes exhibited faster RT than female athletes (β = 0.05, p = 0.049). Another linear age–RT regression model (R² = 0.19, p < 0.001) showed that for every 1-year increase in age, RT increased by 3.6 ms after accounting for event discipline and sex (β = 0.0036, p < 0.001). Event discipline was not significantly associated with RT (p > 0.05). A separate age–RT multiple linear regression model (R² = 0.6, p < 0.01) was conducted, accounting for task, sex, skeletal muscle mass, and body fat percentage. While significant associations occurred between task and RT, sex and RT (all p < 0.01), skeletal muscle mass and percent body fat were not significantly associated with RT (all p > 0.05).

**Fig 4:**
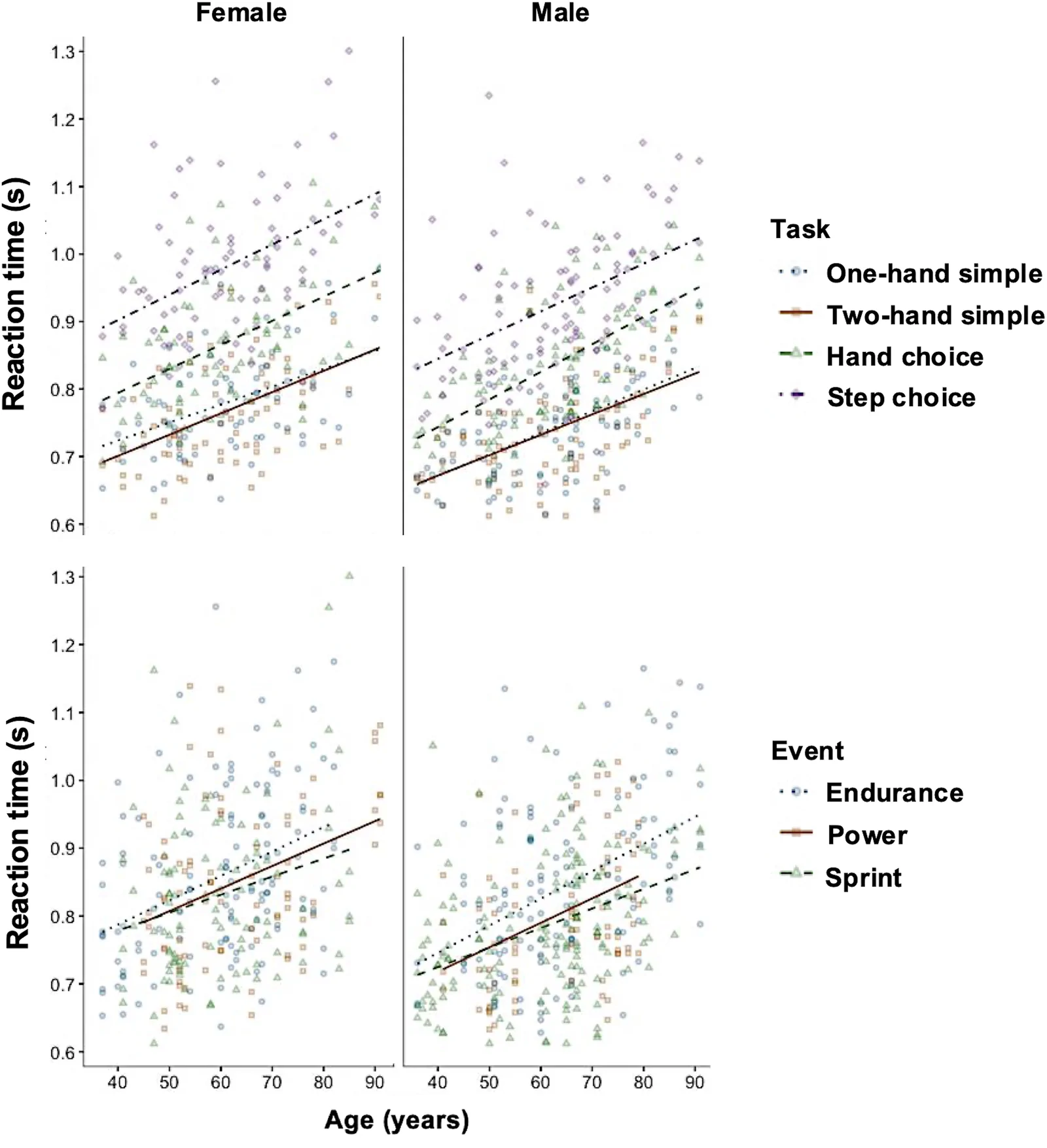
Reaction time stratified by task and sporting discipline in masters athletes.

## Discussion

To our knowledge, this is the first study to investigate how aging, sex, event discipline, and task complexity influence sensorimotor RT in masters athletes spanning a large age range. The main findings were threefold: (1) RT slowed progressively with advancing age across all tasks and in both sexes, with the largest slowing observed in athletes aged ≥80 years; (2) Male athletes demonstrated consistently faster RT than female athletes across all tasks; (3) Sprint athletes showed faster RT than endurance athletes, whereas power athletes did not differ significantly from either group. These findings largely support our a priori hypotheses: RT slowed with advancing age despite lifelong exercise training, with males exhibiting faster RT than females. However, the hypothesis that both sprint and power athletes would outperform endurance athletes was only partially confirmed, as this pattern held for sprint athletes but not for power athletes, who did not differ significantly from either group.

The linear regression analysis revealed that RT slows by ~3.3 ms per year after adjusting for sex, task complexity, and event disciplines. Despite a rate of 3.3 ms per year appearing trivial, it can translate to roughly 100–150 ms of RT slowing if cumulated over three to four decades. This is a magnitude comparable to group differences reported between fallers and non-fallers on choice stepping reaction time (1,322 ± 331 vs. 1,168 ± 203 ms), suggesting that seemingly small annual increments may accumulate into functionally relevant sensorimotor slowing over an athletic career [7]. The correlation analyses also demonstrated moderate to strong positive associations between age and RT across tasks. These results collectively suggest that RT slowing occurs progressively across adulthood and that chronological aging is a major determinant of sensorimotor RT performance even among highly active older adults. A closer examination, however, indicates curvilinear increases in RT only in males for the two-hand simple and step choice tasks, which involve greater bilateral coordination or whole-body responses, placing higher demands on motor planning and neuromuscular control. Such findings suggest that tasks requiring greater coordination or postural integration may be more sensitive to advanced-age declines in neuromuscular function. The discipline subgroup analysis (S2 Fig) suggests that the general male curvature observed in the overall sample may be selectively driven by sprint athletes, whose reliance on fast-twitch motor unit recruitment and explosive neural drive renders them particularly susceptible to the accelerating age-related sensorimotor decline characteristic of older ages. This notion is evident in the observation that the largest and most accelerated slowing is consistently observed in athletes aged >80 years. An interesting observation is that male athletes appear to slow down more sharply in later decades compared with their female peers. Healthy older males exhibit a steeper age-related decline in global mental status, psychomotor speed, and integration than their female counterparts [26]. While not directly measured in the present study, this mechanism may partially explain the accelerated (quadratic) increases in RT observed in men in our study, particularly for tasks requiring rapid sensorimotor integration and decision-making.

The progressive increases in RT with aging observed in masters athletes are consistent with previous large-scale studies in different populations across a wide lifespan [6,17]. RT reflects the integrated function of sensory detection, central information processing, and motor execution (1). Age-related changes in neural conduction velocity, synaptic transmission efficiency, and cortical processing speed have been proposed as candidate mechanisms underlying this decline [27], despite not being tested in the present study. The total grey matter volume, which is involved in central processing, decreases with aging in both men and women [28]. Specifically, the grey matter volume of the sensorimotor cortex and the pre-supplementary motor area, and the white matter of the superior parietal lobule and inferior occipital gyrus, decrease with aging, from the age of 20s to 80s, suggesting cognitive and motor declines with aging [19,29]. It is plausible to hypothesize that these physiological changes take place in masters athletes and contribute to the unavoidable increases in RT. Age-related reductions in sensorimotor performance are more pronounced in information-processing demands than in motor output [30,31]. It is unknown if such a concept can be applied to masters athletes.

Males outperform females in visuospatial abilities, while females display better performance in verbal learning and memory, and fluent language production [26,32,33]. These sex differences have been attributed in prior literature to physiological and neuromuscular factors [34–36] such as muscle mass distribution, neural conduction velocity, or motor unit recruitment, as well as sociocultural factors. Since neuromuscular or neural parameters were not directly measured, these remain candidate explanations rather than confirmed mechanisms in our cohort. We also found that male masters athletes generally demonstrated faster RT than female athletes across different RT tasks, although the magnitude of the sex difference was relatively modest. However, the relations between age and RT were similar in both sexes. Additionally, Fisher’s r-to-z tests indicated no significant differences in correlation strength between sexes. Furthermore, no interaction effects between age and sex were detected in regression models. Such findings suggest that the trajectory of age-related RT slowing occurs at a similar rate in both sexes.

One strength of the present study is the inclusion of a variety of RT tasks. The RT progression from simple hand responses to choice responses and the whole-body stepping task likely reflects increasing cognitive and motor demands. The one-hand simple task includes processing stages of sensory encoding, motor preparation, and motor execution [37]. The two-hand simple task is usually longer than one-hand simple RT, due to effector assignment from both hands in bimanual responses, compared with one hand [38]. It adds a motor selection stage based on the process of the one-hand simple task, when assigning a motor task to one of the two hands, involving activation of one hand and inhibition of the other. Further, the hand and step choice tasks incrementally add stages of stimulus discrimination, response selection, and response inhibition, while focusing on deactivating one light color and neglecting the other color during the task [39]. The increase in RT as task complexity rises suggests a prominent role of higher-order cognitive processing [30,31]. With advancing age, fine control of skeletal muscle gradually deteriorates, a process that has been associated with reductions in brain tissue volume [19]. When the excitability of central processing is compared between young and older adults, older adults require greater excitatory and inhibitory control in motor selection [40]. Additionally, motor units remodel and shift toward larger single motor unit action potentials and a reduced number of smaller units with aging, indicating denervation and reinnervation processes that contribute to diminished fine motor control [41,42]. Indeed, sarcopenia has been associated with slower reaction time [43]. Interestingly, in the present cohort, neither skeletal muscle mass nor percent body fat was significantly associated with RT after adjusting for task and sex, contrasting with prior reports [43] and may reflect that RT in this population is governed more strongly by central neural factors than by peripheral muscular characteristics, though this interpretation remains speculative without direct neuromuscular or neuroimaging measures. The regression model confirmed a significant linear trend across task complexity, indicating that RT progressively increased as tasks required more complex sensorimotor integration. Importantly, no interaction was observed between age and task complexity, suggesting that aging affects both simple and complex reaction processes in a broadly similar manner. In other words, the rate of age-related slowing appears comparable across tasks.

The local remodeling of motor units with aging might be relevant to the faster RT observed in the sprint athletes, who rely heavily on explosive neuromuscular responses and rapid reaction to starting signals. Sprint training requires rapid and repeated activation of high-threshold motor units and may promote more efficient neuromuscular transmission and faster neural drive [44]. This neuromuscular ability could contribute to shorter RT in sprint athletes compared with endurance and power athletes, whose training emphasizes sustained or maximal force production rather than rapid motor unit recruitment. In spite of the repeated exposure to RT tasks in training and competition, age-related changes in RT in sprinters were similar to athletes in other disciplines, suggesting that chronic sprint training does not seem to attenuate age-related changes in RT.

As in any research studies, there are several limitations that should be noted in the present study. First, the sedentary comparison group was not included. To provide insight into potential group differences between masters athletes and non-exercising older adults, we located a previously published study in community-dwelling older adults [24]. In masters athletes and community-dwelling older adults who are matched for the ages (64.4 ± 2.9 vs. 63.3 ± 4.4 years) and BMI (23.1 ± 3.2 vs. 24.0 ± 3.5 kg/m²), RT (step choice RT) was numerically faster (17%) in masters athletes compared with the community-dwelling older adults (0.93 ± 0.09 vs. 1.12 ± 0.15 s), though this comparison should be interpreted cautiously given differences in recruitment setting, population characteristics, and no formal statistical test was performed. Second, a cross-sectional design precludes any causal inference. Third, track and field event discipline classification was based on the primary event discipline, which may not fully capture their mixed training backgrounds, since many masters athletes compete across multiple disciplines and may engage in overlapping neuromuscular and endurance training. Fourth, the RT protocol did not include direct measures of cognitive domains and neuromuscular function, which could help explain the physiological mechanisms underlying slower responses with age. Moreover, RT is a task-specific skill that can be influenced by motor learning independent of broader physiological aging processes. Therefore, the present findings should not be interpreted as a comprehensive marker of overall aging quality. Fifth, the unequal distribution of participants across age groups, particularly in the oldest category (≥80 years group, n = 19), may have reduced statistical power and increased the uncertainty of reaction time estimates for this subgroup. Sixth, as with most studies of masters athletes, the present finding is subject to a “healthy survivor” bias, whereby the oldest competitors represent a highly selected subset of individuals who remained healthy enough to sustain training and competition into advanced age, meaning the true rate of age-related RT decline in the broader aging population is likely underestimated. Finally, data on athletic performance, cardiorespiratory fitness, training history, and years of training, which might be able to explain partial variance in the current analysis, are not available in the present analysis. Future studies should incorporate detailed physiological, cognitive, and training-related measures to clarify the mechanisms underlying age-related RT slowing and to determine whether specific training modalities targeting neuromotor speed or cognitive–motor integration can attenuate this decline in older athletes. Additionally, because our RT measure reflects total response time without disentangling the central processing and mechanical execution components, future studies using instrumentation with a discrete movement-onset sensor would allow RT and movement time to be assessed separately.

## Conclusions

Across all sensorimotor tasks, masters track and field athletes demonstrated relatively preserved reaction time performance, although RT progressively slowed with advancing age and increasing task complexity. Sex differences in RT were consistent across age groups, with a similar rate of age-related slowing observed in males and females. Event discipline had minimal influence on RT, with sprint athletes tending to show faster RT than endurance and power athletes but no discipline-specific difference in the trajectory of age-related change. Overall, these findings suggest that aging remains strongly associated with slower RT even among highly trained older athletes, despite long-term participation in competitive sport.

## Supporting information

S1 Fig Distribution of participants stratified by age and event discipline. (PNG)

S2 Fig Linear and quadratic correlations between reaction time and age stratified by sex and sporting discipline in masters athletes. (PNG)

S1 Table Number of participants by age, sex, and sporting discipline. (DOCX)
