# Supplementary material for: Age-related slowing of sensorimotor reaction time in masters track and field athletes
Source: PLoS One. 2026 Nov 11;21(11):e0360641. doi: 10.1371/journal.pone.0360641 (PMC13754140; doi:10.1371/journal.pone.0360641)
Supplement: Supplementary file 1 [file pone.0360641.s003.docx]

**S1 Table**. Number of participants by age, sex, and sporting discipline.

| **Age group** | **Female** | **Male** | **Total** | **Endurance** | **Power** | **Sprint** |
| --- | --- | --- | --- | --- | --- | --- |
| 35-49 | 16 | 17 | 33 | 12 | 6 | 15 |
| 50-59 | 22 | 21 | 43 | 14 | 12 | 15 |
| 60-69 | 24 | 29 | 53 | 22 | 7 | 24 |
| 70-79 | 13 | 21 | 34 | 10 | 14 | 9 |
| ≥80 | 7 | 12 | 19 | 9 | 2 | 7 |
